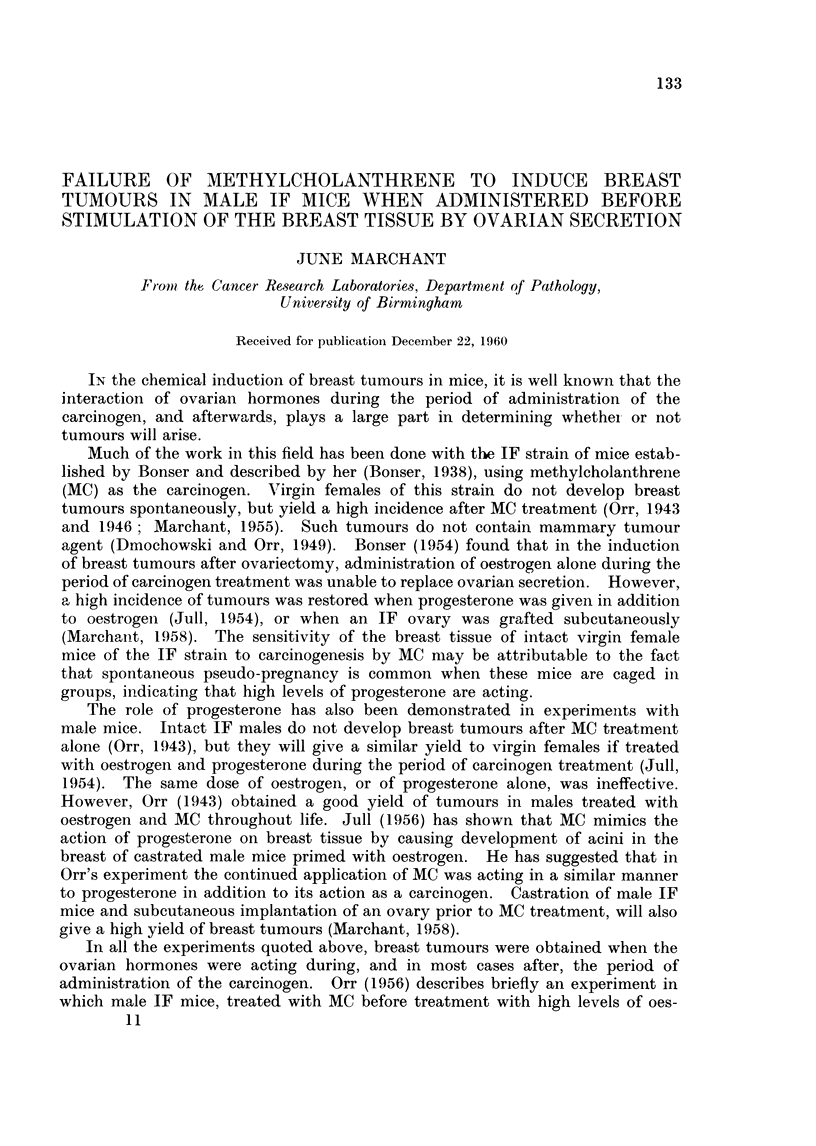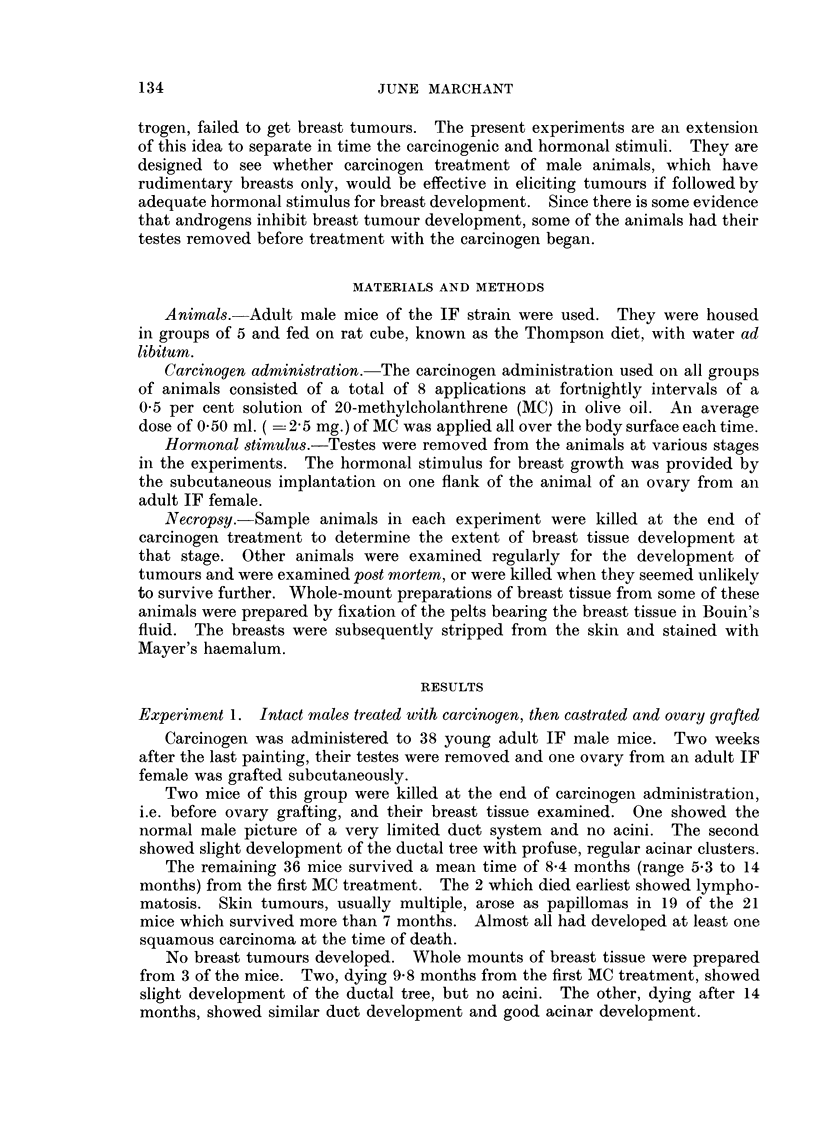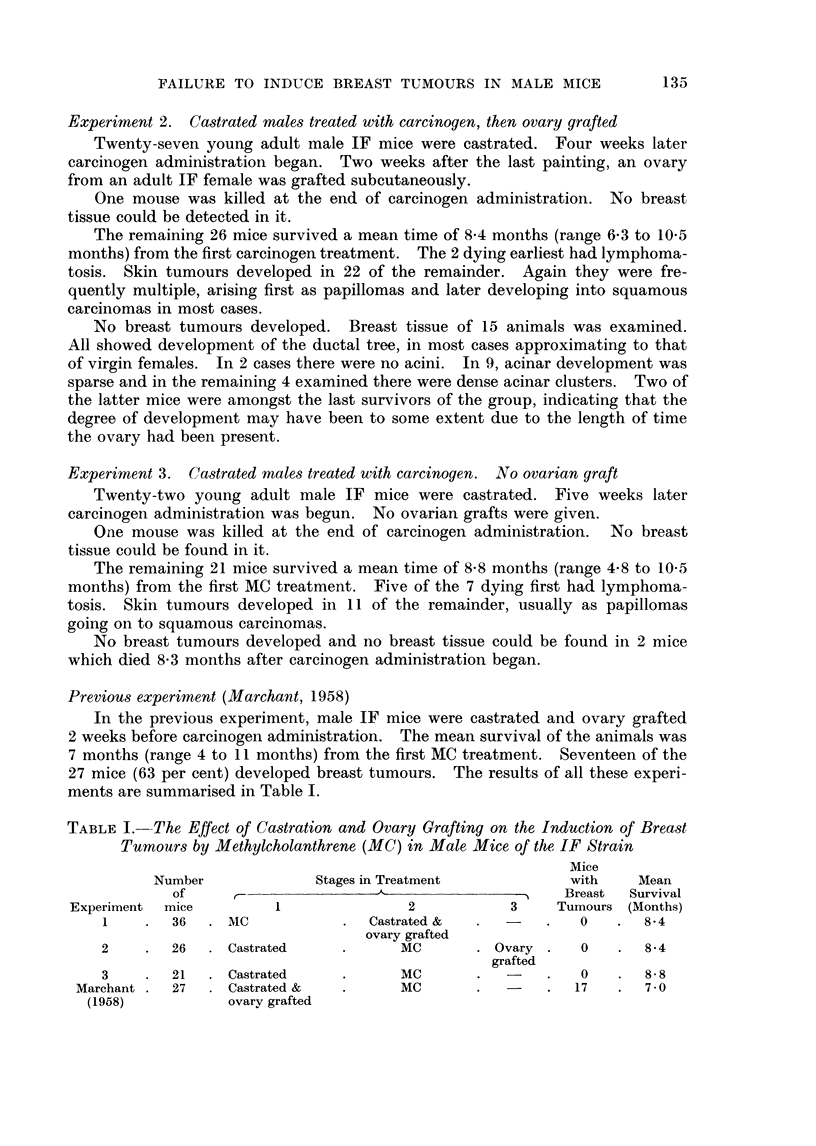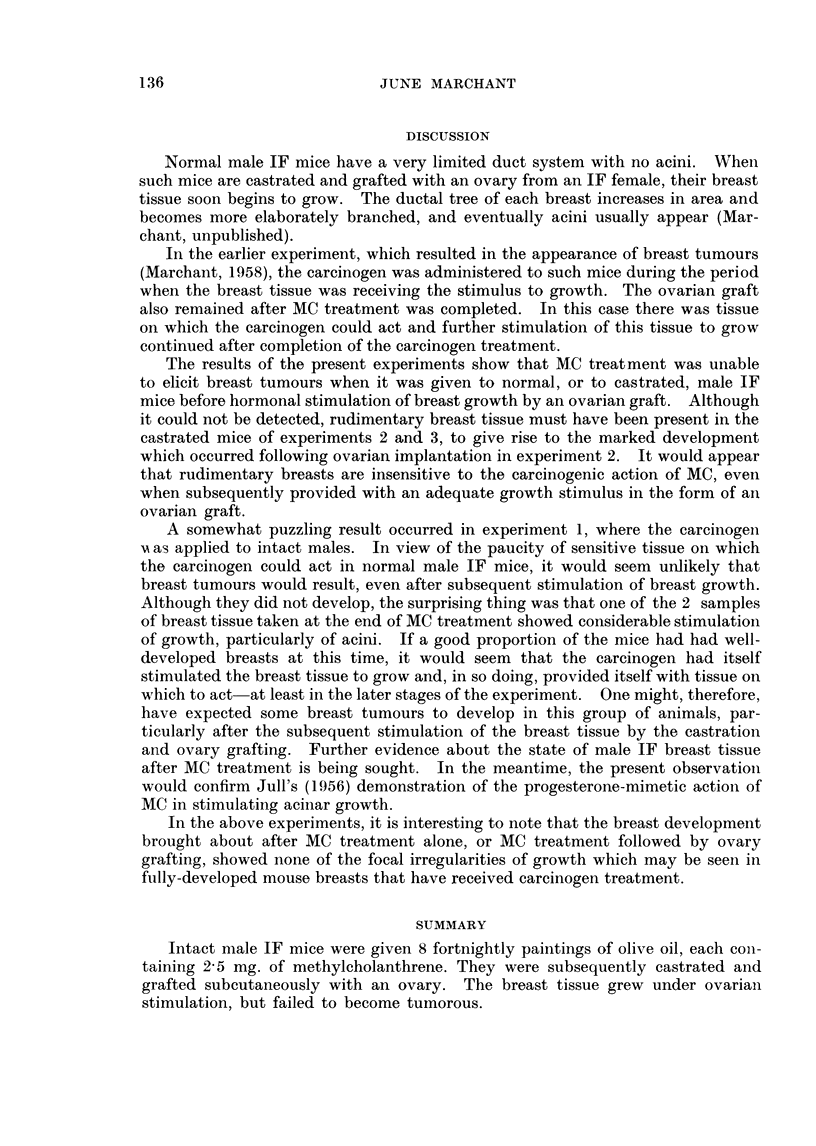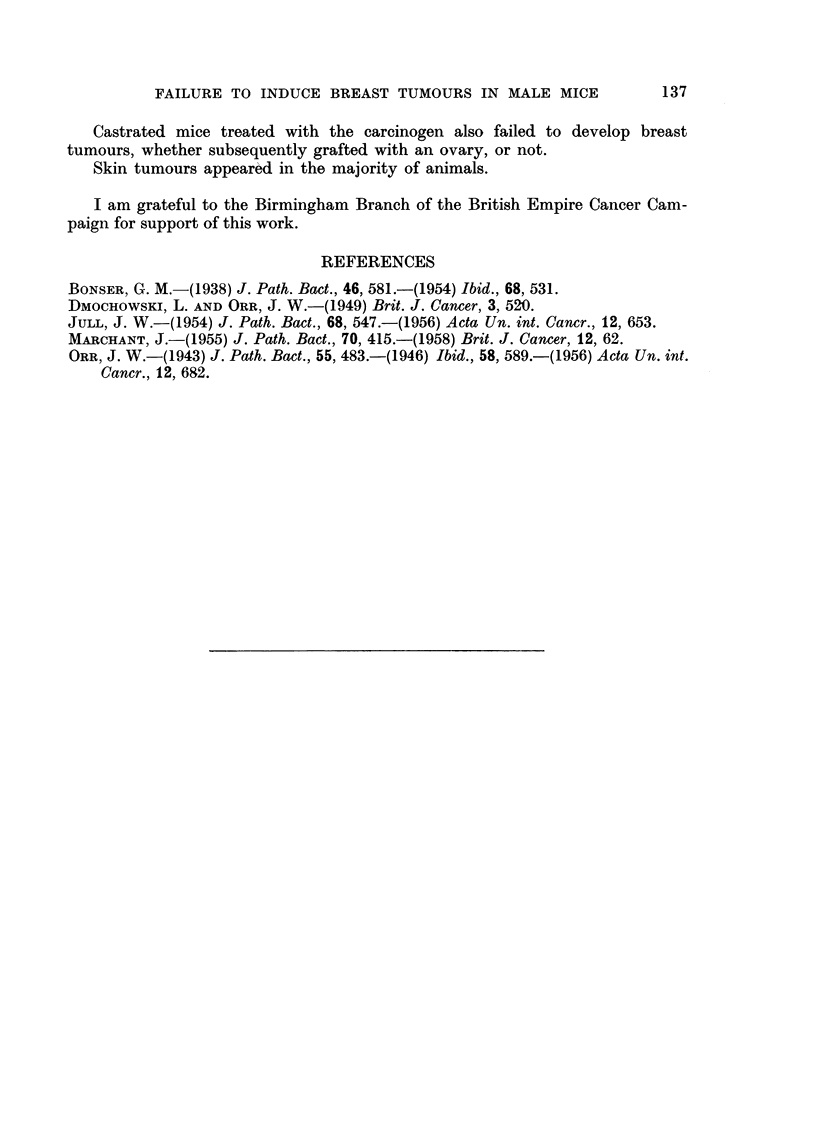# Failure of Methylcholanthrene to Induce Breast Tumours in Male IF Mice When Administered Before Stimulation of the Breast Tissue by Ovarian Secretion

**DOI:** 10.1038/bjc.1961.16

**Published:** 1961-03

**Authors:** June Marchant


### FAILURE OF METHYLCHOLANTHRENE TO INDUCE BREAST TUMOURS IN MALE IF MICE WHEN ADMINISTERED BEFORE STIMULATION OF THE BREAST TISSUE BY OVARIAN SECRETION

JUNE MARCHANT

*From the Cancer Research Laboratories, Department of Pathology, University of Birmingham*

Received for publication December 22, 1960

In the chemical induction of breast tumours in mice, it is well known that the interaction of ovarian hormones during the period of administration of the carcinogen, and afterwards, plays a large part in determining whether or not tumours will arise.

Much of the work in this field has been done with the IF strain of mice established by Bonser and described by her (Bonser, 1938), using methylcholanthrene (MC) as the carcinogen. Virgin females of this strain do not develop breast tumours spontaneously, but yield a high incidence after MC treatment (Orr, 1943 and 1946 ; Marchant, 1955). Such tumours do not contain mammary tumour agent (Dmochowski and Orr, 1949). Bonser (1954) found that in the induction of breast tumours after ovariectomy, administration of oestrogen alone during the period of carcinogen treatment was unable to replace ovarian secretion. However, a high incidence of tumours was restored when progesterone was given in addition to oestrogen (Jull, 1954), or when an IF ovary was grafted subcutaneously (Marchant, 1958). The sensitivity of the breast tissue of intact virgin female mice of the IF strain to carcinogenesis by MC may be attributable to the fact that spontaneous pseudo-pregnancy is common when these mice are caged in groups, indicating that high levels of progesterone are acting.

The role of progesterone has also been demonstrated in experiments with male mice. Intact IF males do not develop breast tumours after MC treatment alone (Orr, 1943), but they will give a similar yield to virgin females if treated with oestrogen and progesterone during the period of carcinogen treatment (Jull, 1954). The same dose of oestrogen, or of progesterone alone, was ineffective. However, Orr (1943) obtained a good yield of tumours in males treated with oestrogen and MC throughout life. Jull (1956) has shown that MC mimics the action of progesterone on breast tissue by causing development of acini in the breast of castrated male mice primed with oestrogen. He has suggested that in Orr's experiment the continued application of MC was acting in a similar manner to progesterone in addition to its action as a carcinogen. Castration of male IF mice and subcutaneous implantation of an ovary prior to MC treatment, will also give a high yield of breast tumours (Marchant, 1958).

In all the experiments quoted above, breast tumours were obtained when the ovarian hormones were acting during, and in most cases after, the period of administration of the carcinogen. Orr (1956) describes briefly an experiment in which male IF mice, treated with MC before treatment with high levels of oestrogen, failed to get breast tumours. The present experiments are an extension of this idea to separate in time the carcinogenic and hormonal stimuli. They are designed to see whether carcinogen treatment of male animals, which have rudimentary breasts only, would be effective in eliciting tumours if followed by adequate hormonal stimulus for breast development. Since there is some evidence that androgens inhibit breast tumour development, some of the animals had their testes removed before treatment with the carcinogen began.

### MATERIALS AND METHODS

*Animals.*—Adult male mice of the IF strain were used. They were housed in groups of 5 and fed on rat cube, known as the Thompson diet, with water *ad libitum.*

*Carcinogen administration.*—The carcinogen administration used on all groups of animals consisted of a total of 8 applications at fortnightly intervals of a 0·5 per cent solution of 20-methylcholanthrene (MC) in olive oil. An average dose of 0·50 ml. ( = 2·5 mg.) of MC was applied all over the body surface each time.

*Hormonal stimulus.*—Testes were removed from the animals at various stages in the experiments. The hormonal stimulus for breast growth was provided by the subcutaneous implantation on one flank of the animal of an ovary from an adult IF female.

*Necropsy.*—Sample animals in each experiment were killed at the end of carcinogen treatment to determine the extent of breast tissue development at that stage. Other animals were examined regularly for the development of tumours and were examined *post mortem*, or were killed when they seemed unlikely to survive further. Whole-mount preparations of breast tissue from some of these animals were prepared by fixation of the pelts bearing the breast tissue in Bouin's fluid. The breasts were subsequently stripped from the skin and stained with Mayer's haemalum.

### RESULTS

### *Experiment* 1. *Intact males treated with carcinogen, then castrated and ovary grafted*

Carcinogen was administered to 38 young adult IF male mice. Two weeks after the last painting, their testes were removed and one ovary from an adult IF female was grafted subcutaneously.

Two mice of this group were killed at the end of carcinogen administration, i.e. before ovary grafting, and their breast tissue examined. One showed the normal male picture of a very limited duct system and no acini. The second showed slight development of the ductal tree with profuse, regular acinar clusters.

The remaining 36 mice survived a mean time of 8·4 months (range 5·3 to 14 months) from the first MC treatment. The 2 which died earliest showed lymphomatosis. Skin tumours, usually multiple, arose as papillomas in 19 of the 21 mice which survived more than 7 months. Almost all had developed at least one squamous carcinoma at the time of death.

No breast tumours developed. Whole mounts of breast tissue were prepared from 3 of the mice. Two, dying 9·8 months from the first MC treatment, showed slight development of the ductal tree, but no acini. The other, dying after 14 months, showed similar duct development and good acinar development.

*Experiment* 2. *Castrated males treated with carcinogen, then ovary grafted*

Twenty-seven young adult male IF mice were castrated. Four weeks later carcinogen administration began. Two weeks after the last painting, an ovary from an adult IF female was grafted subcutaneously.

One mouse was killed at the end of carcinogen administration. No breast tissue could be detected in it.

The remaining 26 mice survived a mean time of 8·4 months (range 6·3 to 10·5 months) from the first carcinogen treatment. The 2 dying earliest had lymphomatosis. Skin tumours developed in 22 of the remainder. Again they were frequently multiple, arising first as papillomas and later developing into squamous carcinomas in most cases.

No breast tumours developed. Breast tissue of 15 animals was examined. All showed development of the ductal tree, in most cases approximating to that of virgin females. In 2 cases there were no acini. In 9, acinar development was sparse and in the remaining 4 examined there were dense acinar clusters. Two of the latter mice were amongst the last survivors of the group, indicating that the degree of development may have been to some extent due to the length of time the ovary had been present.

*Experiment* 3. *Castrated males treated with carcinogen. No ovarian graft*

Twenty-two young adult male IF mice were castrated. Five weeks later carcinogen administration was begun. No ovarian grafts were given.

One mouse was killed at the end of carcinogen administration. No breast tissue could be found in it.

The remaining 21 mice survived a mean time of 8·8 months (range 4·8 to 10·5 months) from the first MC treatment. Five of the 7 dying first had lymphomatosis. Skin tumours developed in 11 of the remainder, usually as papillomas going on to squamous carcinomas.

No breast tumours developed and no breast tissue could be found in 2 mice which died 8·3 months after carcinogen administration began.

*Previous experiment* (*Marchant*, 1958)

In the previous experiment, male IF mice were castrated and ovary grafted 2 weeks before carcinogen administration. The mean survival of the animals was 7 months (range 4 to 11 months) from the first MC treatment. Seventeen of the 27 mice (63 per cent) developed breast tumours. The results of all these experiments are summarised in Table I.

TABLE I.—*The Effect of Castration and Ovary Grafting on the Induction of Breast Tumours by Methylcholanthrene* (*MC*) *in Male Mice of the IF Strain*

| Experiment | Number of mice | Stages in Treatment 1 | 2 | 3 | Mice with Breast Tumours | Mean Survival (Months) |
|---|---|---|---|---|---|---|
| 1 | 36 | MC | Castrated & ovary grafted | — | 0 | 8·4 |
| 2 | 26 | Castrated | MC | Ovary grafted | 0 | 8·4 |
| 3 | 21 | Castrated | MC | — | 0 | 8·8 |
| Marchant (1958) | 27 | Castrated & ovary grafted | MC | — | 17 | 7·0 |

### DISCUSSION

Normal male IF mice have a very limited duct system with no acini. When such mice are castrated and grafted with an ovary from an IF female, their breast tissue soon begins to grow. The ductal tree of each breast increases in area and becomes more elaborately branched, and eventually acini usually appear (Marchant, unpublished).

In the earlier experiment, which resulted in the appearance of breast tumours (Marchant, 1958), the carcinogen was administered to such mice during the period when the breast tissue was receiving the stimulus to growth. The ovarian graft also remained after MC treatment was completed. In this case there was tissue on which the carcinogen could act and further stimulation of this tissue to grow continued after completion of the carcinogen treatment.

The results of the present experiments show that MC treatment was unable to elicit breast tumours when it was given to normal, or to castrated, male IF mice before hormonal stimulation of breast growth by an ovarian graft. Although it could not be detected, rudimentary breast tissue must have been present in the castrated mice of experiments 2 and 3, to give rise to the marked development which occurred following ovarian implantation in experiment 2. It would appear that rudimentary breasts are insensitive to the carcinogenic action of MC, even when subsequently provided with an adequate growth stimulus in the form of an ovarian graft.

A somewhat puzzling result occurred in experiment 1, where the carcinogen was applied to intact males. In view of the paucity of sensitive tissue on which the carcinogen could act in normal male IF mice, it would seem unlikely that breast tumours would result, even after subsequent stimulation of breast growth. Although they did not develop, the surprising thing was that one of the 2 samples of breast tissue taken at the end of MC treatment showed considerable stimulation of growth, particularly of acini. If a good proportion of the mice had had well-developed breasts at this time, it would seem that the carcinogen had itself stimulated the breast tissue to grow and, in so doing, provided itself with tissue on which to act—at least in the later stages of the experiment. One might, therefore, have expected some breast tumours to develop in this group of animals, particularly after the subsequent stimulation of the breast tissue by the castration and ovary grafting. Further evidence about the state of male IF breast tissue after MC treatment is being sought. In the meantime, the present observation would confirm Jull's (1956) demonstration of the progesterone-mimetic action of MC in stimulating acinar growth.

In the above experiments, it is interesting to note that the breast development brought about after MC treatment alone, or MC treatment followed by ovary grafting, showed none of the focal irregularities of growth which may be seen in fully-developed mouse breasts that have received carcinogen treatment.

### SUMMARY

Intact male IF mice were given 8 fortnightly paintings of olive oil, each containing 2·5 mg. of methylcholanthrene. They were subsequently castrated and grafted subcutaneously with an ovary. The breast tissue grew under ovarian stimulation, but failed to become tumorous.

Castrated mice treated with the carcinogen also failed to develop breast tumours, whether subsequently grafted with an ovary, or not.

Skin tumours appeared in the majority of animals.

I am grateful to the Birmingham Branch of the British Empire Cancer Campaign for support of this work.

### REFERENCES

BONSER, G. M.—(1938) *J. Path. Bact.*, **46**, 581.—(1954) *Ibid.*, **68**, 531.
DMOCHOWSKI, L. AND ORR, J. W.—(1949) *Brit. J. Cancer*, **3**, 520.
JULL, J. W.—(1954) *J. Path. Bact.*, **68**, 547.—(1956) *Acta Un. int. Cancr.*, **12**, 653.
MARCHANT, J.—(1955) *J. Path. Bact.*, **70**, 415.—(1958) *Brit. J. Cancer*, **12**, 62.
ORR, J. W.—(1943) *J. Path. Bact.*, **55**, 483.—(1946) *Ibid.*, **58**, 589.—(1956) *Acta Un. int. Cancr.*, **12**, 682.